# PP58 Enhancing Health Technology Assessment Understanding Through Targeted Educational Programs

**DOI:** 10.1017/S0266462324002290

**Published:** 2025-01-07

**Authors:** Elena Chitan, Alina Timotin, Angela Anisei, Ilie Volovei, Oleg Lozan

## Abstract

**Introduction:**

As health technology assessment (HTA) becomes vital in healthcare decision-making, the demand for specialized education grows. A new HTA course, tailored to the specific needs of the Republic of Moldova’s health system , was launched under a joint project—a collaboration between the World Bank, the Swiss Cooperation Office, Radboud University Medical Center, and the School of Public Health Management. The course targets first- and second-year master’s students, and health professionals enrolled in continuing education.

**Methods:**

The course aimed to introduce the fundamentals of HTA. Participants included 49 master’s students and 26 health professionals. A pre- and post-test model was employed, with participants completing a 10-(multiple) question HTA knowledge assessment at both the start and end of the course.

**Results:**

Initial pre-test results showed an average score of 30 percent, reflecting limited prior HTA knowledge. Following course completion, the post-test average escalated to 80 percent. This 50 percent increase in knowledge was consistent across student and professional groups. While a significant 79 percent of participants accurately answered questions about the use of HTA reports from other jurisdictions, identification of stakeholders, and the elements of establishing PICO (population, intervention, comparator, outcome), they found it more challenging to understand the deeper aspects of HTA. Notably, about 72 percent had difficulties with questions related to the main goals of HTA, evaluating the broader impacts of health technologies, and starting the evidence-based deliberative decision-making process.

**Conclusions:**

The HTA course successfully covered basic concepts, yet it also highlighted the need for more comprehensive teaching of complex topics. The participants showed varying levels of understanding. This underscores the necessity for an HTA curriculum that equally emphasizes fundamental knowledge and in-depth analysis, preparing future healthcare professionals for complex decision-making in their roles.

